# Increased HERV-K(HML-2) Transcript Levels Correlate with Clinical Parameters of Liver Damage in Hepatitis C Patients

**DOI:** 10.3390/cells10040774

**Published:** 2021-03-31

**Authors:** Melanie Weber, Vidya Padmanabhan Nair, Tanja Bauer, Martin F. Sprinzl, Ulrike Protzer, Michelle Vincendeau

**Affiliations:** 1 Institute of Virology, HelmholtzZentrum München, Neuherberg 85764, Germany; weber.m.melanie@gmail.com (M.W.); vidya.padmanabhan@helmholtz-muenchen.de (V.P.N.); tanja.bauer@helmholtz-muenchen.de (T.B.); 2 Institute of Virology, Technische Universität München, Munich 81675, Germany; 3 German Center for Infection Research (DZIF), Partner Site, Munich 81675, Germany; 4 Medical Department, University Hospital Mainz, Mainz 55131, Germany; Martin.Sprinzl@unimedizin-mainz.de

**Keywords:** hepatitis C virus, human endogenous retroviruses, liver cirrhosis, albumin, viral clearance, direct-acting antivirals

## Abstract

Chronic hepatitis C virus (HCV) infection is closely associated with a plethora of diseases, including cancers and autoimmune disorders. However, the distinct triggers and cellular networks leading to such HCV-derived diseases are poorly understood. Around 8% of the human genome consists of human endogenous retroviruses. They are usually silenced but can be reactivated by environmental conditions, including viral infections. Our current understanding indicates that the activation of one specific family—namely, HERV-K(HML-2)—is linked to distinct pathologies, including cancer and autoimmunity. In this study, we analyzed the transcription levels of HERV-K(HML-2) in 42 HCV-infected patients receiving direct-acting antiviral therapies. Samples from the start of treatment until 12 weeks post-treatment were investigated. Our results show increased HERV-K(HML-2) transcript levels in patients with HCV-derived liver cirrhosis throughout the observation period. Several clinical parameters specifying poor liver function are positively correlated with HERV-K(HML-2) expression. Of note, patients without a sustained viral clearance showed a drastic increase in HERV-K(HML-2) transcript levels. Together, our data suggest that increased HERV-K(HML-2) expression is correlated with reduced liver function as well as therapy success in HCV-infected patients.

## 1. Introduction

Around 71 million people worldwide are chronically infected with hepatitis C. With the development of direct-acting antivirals (DAAs), sustained viral response rates of >90% could be achieved [1,2]. However, hepatitis C virus (HCV) infection remains a risk factor for the development of liver cirrhosis and hepatocellular carcinoma (HCC). Moreover, several other diseases such as neurocognitive disorders or mixed cryoglobulinemia [3] are associated with the infection of persistent, exogenous viruses such as HCV. However, the exact mechanisms driving these related diseases are still not completely understood.

Interestingly, endogenous retroviral elements (ERVs), which make up to 8% of the human genome with over 500,000 copies distributed over all chromosomes [4], are strongly associated with these diseases [5]. HERVs are relics of infectious retroviruses in our ancestors, whose proviruses invaded the germline and expanded via retrotransposition and reinfection [6,7]. Most HERVs have been silenced by mutations and/or epigenetic control mechanisms [8]. However, they can be reactivated by environmental conditions such as exogenous viruses, including HIV-1 [9], and are potentially pathogenic, as they hold the capacity to modify the nuclear genome [10,11]. Retrovirus-like LTRs represent a major population in the human genome that can be transcriptionally active and thus have evolved complex transcriptional enhancers and promoters that allow their transcription in a wide range of tissues and cell types. The analysis of expressed sequence tag (EST) databases show that at least half of the LTRs in the human genome show promoter activity and therefore are part of the cellular transcriptome [12]. Moreover, several HERV groups are still able to code for retroviral proteins and peptides [13] that can be pathological.

The HERV envelope protein is associated with several chronic diseases (autoimmune disease), neurological diseases, and the suppression or stimulation of the immune response [5,14,15]. Some HERV proviruses such as HERV-K(HML-2) express a spliced RNA encoding for proteins such as Rec or NP9 which have been implicated in oncogenesis [16,17]. HERV-K(HML-2) has also been shown to induce neuronal cell death [18,19,20] and reverse transcriptase activity could be detected in the cerebrospinal fluid of amyotrophic lateral sclerosis patients [21]. Additionally, an association between HERV-K(HML-2) and hepatocellular carcinoma (HCC) has been identified [22,23]. Elevated HERV-K(HML-2) levels could be observed in HCC tissue compared to normal tissues, as well as the poorer overall survival of patients with a high HERV-K(HML-2) expression [24]. In addition, an increased expression of the HERV-K(HML-2) as well as HERV-H polymerase has been identified in patients with chronic HCV infection. This remained unchanged after viral clearance using direct-acting antiviral (DAA) treatment [23].

Our study aimed to investigate HERV-K(HML-2) transcriptional levels and their correlation to the pathogenesis of HCV infection using clinical parameters such as liver cirrhosis. We investigated 42 patients for up to 24 weeks of DAA treatment as well as 12 weeks after the end of treatment. Our results show that HERV-K(HML-2) levels are strongly associated with albumin levels and the presence of liver cirrhosis in HCV-positive patients. Moreover, HERV-K(HML-2) levels were increased in patients who did not achieve a sustained viral response.

## 2. Materials and Methods

### 2.1. Isolation of Peripheral Blood Mononuclear Cells (PBMC)

Heparinized whole blood was collected by venipuncture and stored at room temperature. Within 8 h, PBMC isolation was performed. After dilution (1:2) with PBS, whole blood was carefully transferred to 15 mL of Ficoll–Paque solution. A Ficoll density gradient was created by centrifugation (800 g, 15 min, 21 °C, brakes off). The PBMC were separated and washed twice with RPMI-1640 (250 g, 10 min, 21 °C). Turk’s solution was used as a stain to induce erythrocyte lysis for live/dead discrimination, and viable PBMC were then counted under a light microscope.

### 2.2. Cryoconservation and Thawing of PBMC

For cryopreservation, the PBMC were re-suspended in a freezing medium containing FBS and 10% DMSO at a concentration of 1 × 10^6^ cells per milliliter. Subsequently, the PBMC were slowly frozen inside a controlled-grade freezing container at −80 °C. The PBMC stored in nitrogen were thawed in a water bath.

### 2.3. Isolation of RNA Using a Trizol Gradient

The PBMC were thawed and centrifuged (1200 rpm, 10 min, 21 °C). Supernatant was discarded. A total of 1 mL of Trizol (Thermofisher, Waltham, MA, USA) was added to the pellet and the RNA was isolated following the manufacturer’s instructions. The RNA was re-suspended in 30 μL of RNAse-free water. The RNA concentration and quality were verified by Nanodrop and the RNA was stored at −80 °C.

### 2.4. DNAse Treatment

To remove genomic DNA contamination, the RNA samples (1 μg) were treated with 1 U/μg RNase-free RQ1DNase (Promega, Mannheim, Germany), following the manufacturer’s protocol.

### 2.5. First Strand cDNA Synthesis Using Random Hexamer Primers

The reverse transcription of the RNA was generated from 1 μg of total RNA using Superscript II (Thermofisher, Waltham, MA, USA) according to the manufacturer’s protocol using random hexamers.

### 2.6. Quantitative Real-Time PCR

The amplification of *pol*(RT) sequences of the HERV-K(HML-2) family was performed as described in [9]. The amplification of IP10 was performed as described in [25]. Quantitative real-time RT-PCR was performed with the Roche LightCycler 480 System, using LC480 DNA Master SYBR Green and standard LightCycler protocol (Roche Diagnostics, Mannheim, Germany). The RNA-Polymerase II-transcripts (RPII) were analyzed as internal standard, using the primers given in [9].

### 2.7. One-Step Quantitative Real-Time PCR

For some samples with very low yields of RNA concentration, qRT-PCR using KAPA SYBR FAST One-Step (Sigma, Taufkirchen, Germany) was implemented following the manufacturer’s protocol and using the primers described in [9].

### 2.8. Analysis of Quantitative Real-Time PCR

ΔC_T_-values were calculated as follows: ΔC_T_ = C_T_ (gene of interest) − C_T_ (housekeeping gene). The x-fold induction of the experimental parameters relative to RNA Polymerase II was calculated by the 2 ΔC_T_ method [26].

### 2.9. Statistical Analysis

SPSS version 26 was used to complete the statistical analysis and SPSS version 26 as well as Graph Pad Prism 9 were used to generate graphs. The level of significance was set to 5%. Thus, *p*-values of <0.05 were considered statistically significant and *p* < 0.01 was considered highly significant. Bonferroni correction was applied as a post-hoc test whenever pairwise comparisons or associations between more than two groups or time points were calculated. For pairwise comparison, the independent samples *t*-test was used for normally distributed data and Mann-Whitney U was used for non-parametric data. To analyze if data for two different time points within the same subgroup significantly differed from each other, the paired samples t-test was used for normally distributed data and the Wilcoxon signed-rank test was used for data that did not show a normal distribution.

To interpret the development of the parameters over time, a one-way analysis of variance (ANOVA) was chosen for parameters that were normally distributed. For experimental parameters which were not distributed normally, a mixed-model analysis was implemented. The subjects were defined by Patient ID and the time points were the repeated measures. The repeated covariance type was set as unstructured. Restricted maximum likelihood and Satterthwaite approximation were used. The CI was set to 95%. Time points were set as fixed effects. No covariates were taken into account, as neither gender, age, nor liver cirrhosis had a significant influence on the fit of the curve. Associations between binary variables were evaluated using the chi-square test and the results were converted into Cramer’s V for easier interpretation. As the cohort cannot be seen as representative of the population, the calculation of the odds ratios was chosen over the risk ratios. For correlations between the experimental and clinical data, a method to detect associations between parameters with a non-parametrical distribution had to be used, as the experimental data were not distributed normally. Kendall’s tau, also known as Kendall’s Rank Correlation Coefficient, was chosen over Spearman’s rho, as it is less sensitive to the overestimation of associations since its calculations are based on concordant and discordant pairs, while Spearman’s rho relies on deviations.

## 3. Results

### 3.1. Cohort Information

**Figure 1:**
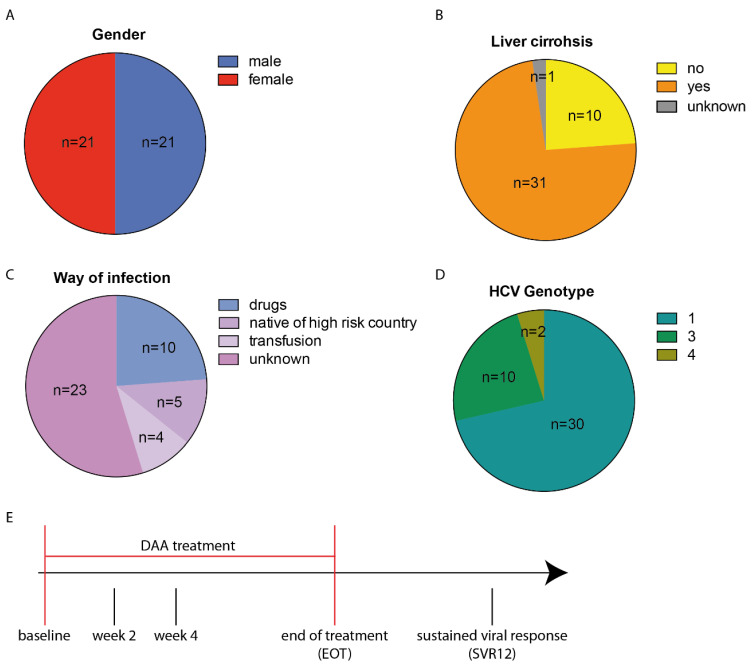
Distribution of descriptive parameters throughout the patient population (*n* = 42) and scheme of time points where clinical samples and data were collected. Chart pies display (**A**) gender distribution, (**B**) liver cirrhosis, (**C**) means of HCV infection as well as (**D**) HCV genotype. (**E**) DAA combination therapy was first administered at the baseline. At EOT treatment ended and 12 weeks later, patients were screened for sustained viral response (SVR12).

A cohort of 42 hepatitis C patients with an equal gender distribution (50% male, 50% female) (Figure 1A) and a median age of 54 years (age range 34–74 years) were treated with DAA combination therapy (Table 1). Liver cirrhosis was present in 31 patients (Figure 1B). The means of infection was unknown in 23 patients, 10 patients were infected via intravenous drug abuse, 4 patients were infected via blood transfusion, and 5 patients were born in a high-risk country for hepatitis C (Figure 1C). Genotype 1 was the most prevalent (*n* = 30), followed by genotype 3 (*n* = 10) and 4 (*n* = 2) (Figure 1D). Clinical parameters as well as the transcript levels of HERV-K(HML2) and quantitative HCV RNA were monitored at five time points before, during, and after DAA treatment. The start of treatment was set as the baseline. Two and four weeks after the start of treatment and at the end of treatment (EOT), as well as 12 weeks after completion, also referred to as sustained viral response (SVR12), the same parameters were collected (Figure 1E).

### 3.2. DAA Treatment Results in Decreased HCV RNA Levels

**Figure 2:**
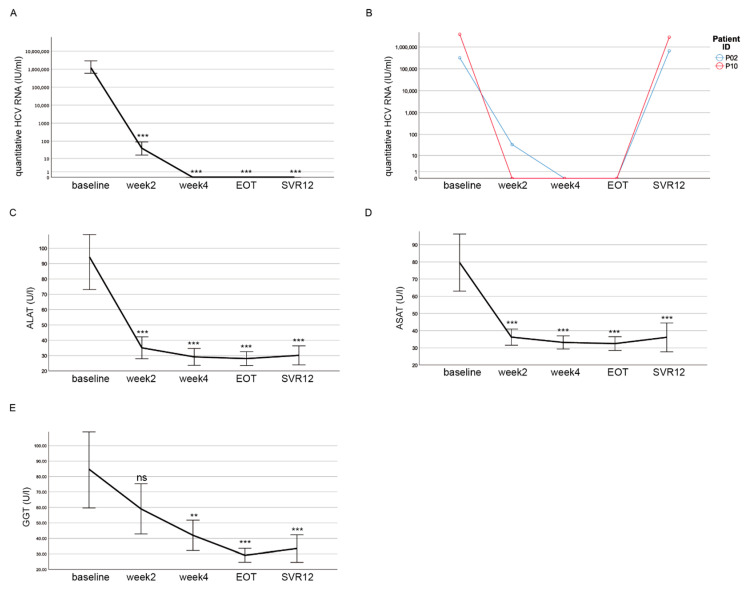
HCV RNA levels decline and liver function normalizes during DAA treatment. (**A**) There was a decline in quantitative HCV RNA after the start of the DAA treatment. At EOT, no HCV RNA was detectable for any patient. (**B**) In two patients, HCV RNA was detected again at levels observed before treatment at SVR12. (**C**–**E**) Decline of ALAT (**C**), ASAT (**D**) and GGT (**E**) activities over the course of DAA treatment. (**A**–**E**) Mean values with the respective 95% confidence interval depicted as error bars are shown for each time point. Significances were related to the baseline and calculated using Bonferroni as a post-hoc test (ns = non significant; ** *p* < 0.01; *** *p* < 0.001).

Throughout the cohort, the HCV RNA levels significantly declined after the start of DAA treatment (Figure 2A). After four weeks of treatment, the HCV RNA levels were not detectable anymore except in one patient. At EOT, the HCV RNA levels were not detectable in any patient (Figure 2A). However, in two patients the HCV RNA levels could again be detected after 12 weeks, suggesting a lack of viral clearance upon DAA treatment at SVR12 (Figure 2B). One patient died during treatment. Thus, a sustained viral response of 92.9% (39 of 42 patients) could be achieved in this cohort. We investigated all the patients for clinical parameters indicating liver damage. The alanine amino transferase (ALAT) (*p* < 0.001) (Figure 2C), aspartate amino transferase (ASAT) (*p* < 0.001) (Figure 2D), and Gamma-Glutamyl-Transferase (GGT) (*p* < 0.001) (Figure 2E) activities significantly decreased in our cohort between the start and EOT and remained low until SVR12. Overall, the DAA treatment was successful in most of the patients, resulting in decreased HCV RNA levels and the improvement of liver function.

### 3.3. HERV-K(HML-2) Transcript Levels Are Increased in Patients with Liver Cirrhosis

**Figure 3:**
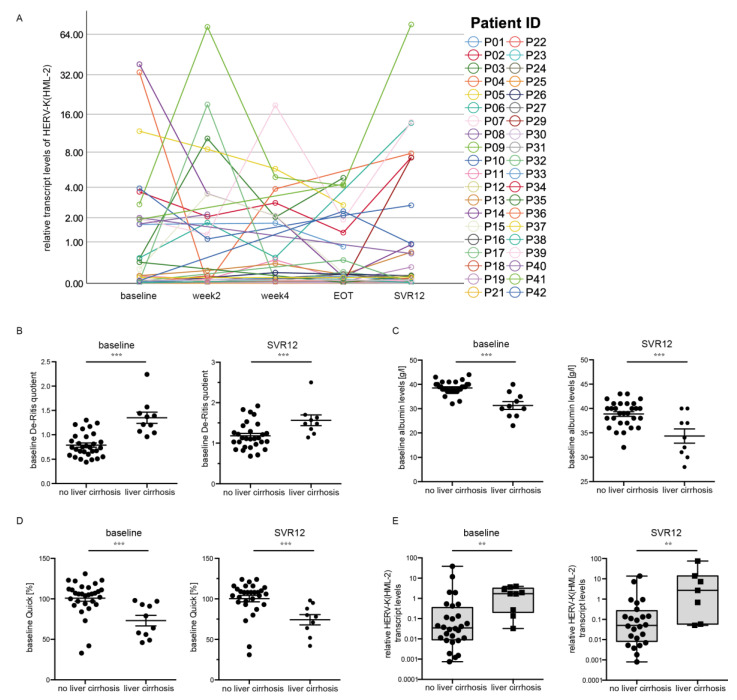
HERV-K(HML-2) transcript levels correlate with liver cirrhosis. (**A**) An increase in HERV-K(HML-2) transcript levels between EOT and SVR12 was detected. (**B**) De Ritis quotient (*p* < 0.001) in patients with and without liver cirrhosis at baseline as well as SVR12. (**C**) Albumin levels (*p* = 0.001) in patients with and without liver cirrhosis at baseline as well as SVR12. (**D**) Quick levels (*p* = 0.001) in patients with and without liver cirrhosis at baseline as well as SVR12. (**E**) Boxplots of HERV-K(HML-2) transcript levels at baseline and SVR12 relative to the housekeeping gene RNA Polymerase II in patients with and without liver cirrhosis are depicted on a logarithmic scale. Dots represent single data points. Significances were analyzed using mixed models (** *p* < 0.01; *** *p* < 0.001).

Interestingly, we could not detect a significant difference in the HERV-K(HML-2) transcript levels during the course of DAA treatment (Figure 3A). However, we found an increase in the HERV-K(HML-2) levels between EOT and SVR12 (mixed-model analysis, *p* = 0.039) (Figure 3A). Patients were divided into two groups (with liver cirrhosis and without liver cirrhosis) to investigate whether the HERV-K(HML-2) expression correlates with clinical parameters associated with worse liver function upon HCV infection. In both groups, we first analyzed the following three clinical parameters: the De Ritis quotient, albumin levels, and Quick levels, all providing clues to liver function. The De Ritis quotient indicates the ratio of the liver enzymes aspartate aminotransferase (ASAT or GOT) and alanine aminotransferase (ALAT or GPT) [27]. Thus, a small De Ritis quotient (<1) indicates minor liver damage, whereas a large quotient (>1) indicates more severe liver damage. We could observe that patients with liver cirrhosis had a higher De Ritis quotient (*p* < 0.001) at the baseline as well as SVR12 (Figure 3B), indicating reduced liver function. Moreover, we analyzed albumin levels. It has been shown that advanced cirrhosis is associated with a decrease in plasmatic albumin [28], thus it is an accepted clinical indicator for liver function. Patients with liver cirrhosis had lower albumin levels (*p* = 0.001) at baseline and SVR12 (Figure 3C), demonstrating impaired liver function in these patients. Additionally, the Quick levels (*p* = 0.001) at baseline as well as SVR12 (Figure 3D) were decreased in patients with liver cirrhosis compared to patients without liver cirrhosis. The Quick level is a laboratory medical parameter of the functional performance of the extrinsic system of blood coagulation. It can be determined on the basis of the measured thromboplastin time [29,30]. Therefore, decreased Quick levels indicate a synthesis disorder of coagulation factors, which in turn indicates a disorder of liver function [27].

Interestingly, the median HERV-K(HML-2) transcript levels at baseline (*p* = 0.003, *n* = 37) and SVR12 (*p* = 0.009, *n* = 31) (Figure 3E) were significantly higher in patients suffering from liver cirrhosis compared to patients without liver cirrhosis. The data indicate a correlation between increased HERV-K(HML-2) transcript levels and worse liver function in patients with HCV infection.

### 3.4. HERV-K(HML-2) Transcript Levels Are Elevated in Patients with Low Albumin Levels

**Figure 4:**
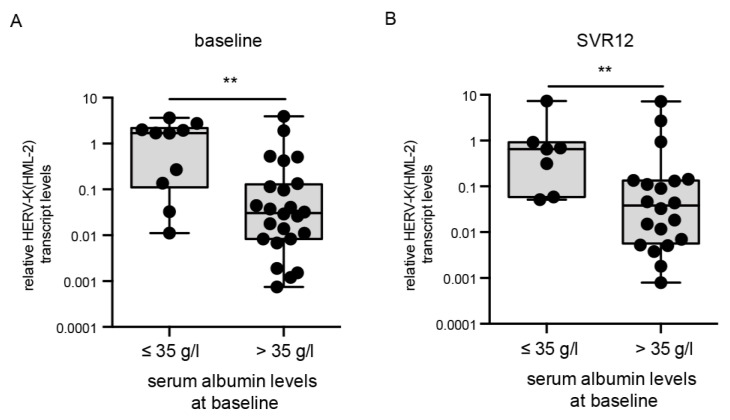
Correlation of HERV-K(HML-2) transcript levels with albumin levels at baseline and SVR12. HERV-K(HML-2) transcript levels were significantly higher in patients with baseline serum albumin levels of ≤ 35 g/L at baseline (*p* = 0.004) as well as SVR12 (*p* = 0.006). Boxplots of HERV-K(HML-2) transcript levels at baseline (**A**) and SVR12 (**B**) relative to the housekeeping gene RNA Polymerase II in patients with low or high albumin levels are depicted on a logarithmic scale. Dots represent a single patient’s data points. Significances were analyzed using mixed models (** *p* < 0.01).

Albumin is a serum-binding protein and has a number of key functions. The functions of albumin include, for example, the transport of various substances such as hormones and fatty acids [31]. Low albumin levels have been associated with advanced liver disease as well as inflammatory diseases [31]. The normal range of albumin is 35–55 g/liter in healthy humans. Throughout the cohort, HERV-K(HML-2) transcript levels at baseline were significantly higher in patients with low albumin levels (≤35 g/L, *p* = 0.004, *n* = 36) (Figure 4A). A statistically significant difference in increased HERV-K(HML-2) transcript levels in patients with low albumin levels (≤35 g/L) could also be detected at SVR12 (*p* = 0.006, *n* = 30) (Figure 4B). At all time points, the HERV-K(HML-2) expression strongly correlated with albumin levels (Table 2). Over the course of DAA treatment, patients with high albumin levels (>35 g/L) showed decreased HERV-K(HML-2) transcript levels (<1), whereas patients with low or unchanged albumin levels (≤35 g/L) showed increased HERV-K(HML-2) expression (>1) (Table 2). HERV-K(HML-2) transcript levels <1 represent lower relative expression than the housekeeping gene RNA-Polymerase II, whereas HERV-K(HML-2) transcript levels >1 signify higher relative expression then the housekeeping gene RNA-Polymerase II. Moreover, the albumin levels recovered towards a mean level within a normal range throughout DAA treatment in these patients. Thus, increased HERV-K(HML-2) expression correlates with low albumin levels at all time points during DAA treatment in HCV-infected patients, once again connecting HERV-K(HML-2) expression with reduced liver function.

### 3.5. The Inflammatory Marker Interferon γ-Inducible Protein 10 (IP 10) Decreases during DAA Treatment and Does Not Correlate with HERV-K(HML-2) Transcript Levels

**Figure 5:**
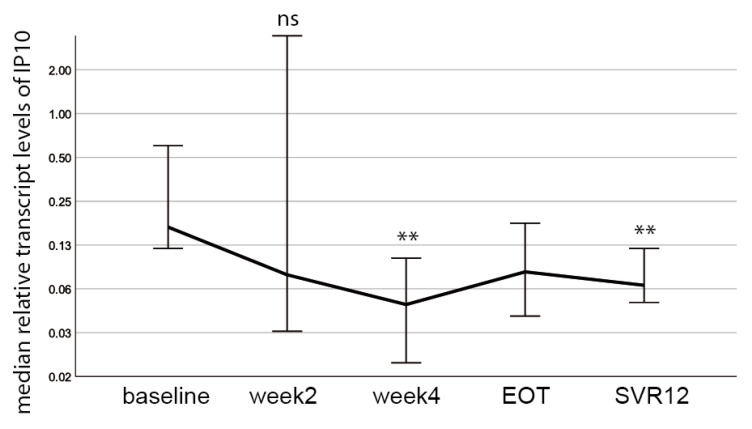
IP10 transcript levels are reduced during DAA treatment. A decrease in median IP10 levels at week 4 (*p* = 0.004) and SVR12 (*p* = 0.003) compared to baseline was detected. Significances were calculated using the Wilcoxon-test and Bonferroni-correction (ns = non significant, ** *p* < 0.01).

To investigate if changes in the HERV-K(HML-2) transcript levels were due to inflammatory responses, we measured the expression of the inflammatory marker interferon γ-inducible protein 10 (IP 10) in all patient samples during the course of DAA treatment using qRT-PCR. We chose the inflammatory maker IP10 as it had already been described serving as prognostic marker for treatment efficacy in chronic HCV-infected patients subjected to DAA therapy [25,32]. Importantly, we observed a significant decrease in IP10 levels at week four and SVR12 compared to baseline (Figure 5), similar to the observed decrease in HCV RNA levels (Figure 2A). However, HERV-K(HML-2) transcript levels (Figure 3A) do not correlate with IP10 levels (Figure 5) or HCV viral RNA levels (Figure 2A). These results suggest that HERV-K(HML-2) expression is not solely driven by inflammatory signaling pathways, which are activated during HCV-infection and reduced upon DAA treatment.

### 3.6. HERV-K(HML-2) Transcript Levels Are Increased in Patients without Sustained Viral Response

**Figure 6:**
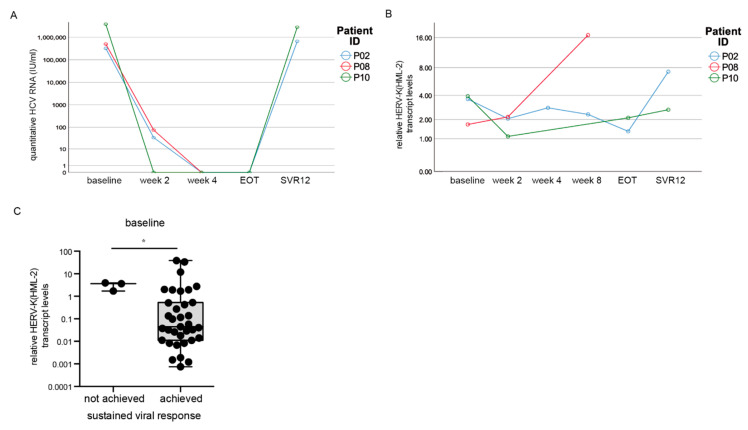
HERV-K(HML-2) levels over time in patients without treatment success. (**A**) HCV RNA progression in three patients without sustained viral clearance. (**B**) HERV-K(HML-2) expression was drastically increased in the last measured point of time. (**C**) At baseline, HERV-K(HML-2) transcript levels were increased in patients without viral clearance compared to patients with sustained viral response. Significances were analyzed using mixed models (* *p* < 0.05).

Two patients (P02, P10) showed a relapse of quantitative HCV RNA at SVR 12 and thus did not achieve a sustained viral response (Figure 2B). Another patient (P08) died after week eight due to a cardiovascular event. All three patients were infected with HCV genotype 1 and had liver cirrhosis. In the beginning, all three patients responded to DAA treatment as their HCV viral load declined and was not detectable at weeks two and four, respectively, upon DAA treatment (Figure 6A). Also at EOT, HCV RNA levels were not detectable for P02 and P10. However, at SVR 12, mean levels of HCV RNA were significantly increased compared to the rest of the cohort (*p* < 0.001) (Figure 6B). In particular, when we compared the HERV-K(HML-2) expression between the last two time points measured, we could detect a drastic increase in the HERV-K(HML-2) transcript levels in all three patients (Figure 6B). In patient P02 as well as P10, we could observe an increase in HERV-K(HML-2) transcript levels at SVR12 compared to EOT. In the case of patient P08, the incline in the HERV-K(HML-2) transcript levels was particularly pronounced at week four (before death) compared to week two. Moreover, at baseline, the HERV-K(HML-2) expression levels were significantly higher in all three patients without successful treatment compared to the rest of the cohort with sustained viral response (*p* = 0.029, *n* = 38) (Figure 6C). However, due to the small number of DAA non-responders, further patients who failed sustained viral clearance, need to be evaluated in future studies. Nevertheless, this side observation gives a hint that increased HERV-K(HML-2) transcript levels might indicate DAA therapy failure.

## 4. Discussion

Our study demonstrates that the expression of one specific HERV family, namely HERV-K(HML-2), correlates with liver cirrhosis as well as clinical parameters indicating impaired liver function, such as low albumin levels, in a cohort of HCV-infected patients treated with direct-acting antiviral (DAA) treatment. Moreover, we found significantly higher HERV-K(HML-2) transcript levels in three patients, who did not achieve sustained viral response upon DAA treatment compared to the rest of the cohort with sustained viral clearance.

Studies in recent years have provided compelling evidence that HERVs critically influence genome functions, mainly through transcriptional control [12,33]. They are now suggested as an important force in the genome evolution and adaptation of an organism to altered environmental conditions and have been associated with several diseases, including cancers and neurodegenerative disorders [34,35]. Multiple human HERV-K(HML-2) proviruses encode for the viral genes, *pol, gag,* and *env* [17]. Besides *pol, gag,* and *env*-encoded proteins, two accessory proteins with regulatory functions are also produced, namely Rec and Np9 [17], which have often been linked to tumor genesis [17,36]. Beside the capacity to encode for full length viral proteins, the human genome comprises ~2000 copies of solitary HERV-K(HML-2) LTRs [37]. These retrovirus-like LTRs have evolved complex transcriptional enhancers and promoters that allow their transcription in a wide range of tissues and cell types. Thus, they possess the potential to impact the expression of genes and even gene networks in our genome [12,38]. To this end, an increased expression of HERV-K(HML-2) upon HCV infection might result in the expression of viral proteins or changes in host gene expression, contributing to the development of cancers and autoimmune disorders [20,39]. However, further detailed functional analyses need to be performed to clarify the exact functional role of specific HERV-K(HML-2) elements on HCV pathogenesis.

Usually, HERV gene expression is controlled epigenetically, e.g., by the methylation of DNA. However, they can be reactivated by environmental conditions, such as infectious agents including HIV-1 or HCV [9,23]. Additionally, inflammatory signaling pathways, as for example NF-κB or NFAT signaling, have been described to increase the expression of certain HERV families [9]. HCV infection can also trigger inflammatory signaling pathways, which diminish during anti-HCV treatment and in turn could impact HERV expression. Interestingly, in our study we observe a significant decrease in the inflammatory marker interferon γ-inducible protein 10 (IP10) during the course of DAA therapy at week four as well as SVR12. HERV-K(HML-2) transcript levels did not change during the course of DAA treatment, which is consistent with recently published data [23]. This suggests that inflammatory pathways do not solely render HERV-K(HML-2) expression in this study. The only significant change in HERV-K(HML-2) expression could be observed between EOT and SVR12. As DAA treatment was stopped at EOT, it is hard to say if the observed rebound in HERV-K(HML-2) expression is related to HCV pathogenesis or affected by the end of antiviral therapy. Future studies will be needed to clarify this interesting aspect.

As liver biopsies are not easy to obtain, because it is an invasive procedure associated with pain and complications and therefore not suitable for routine clinical diagnostics, we used state-of-the-art, non-invasive blood makers (including ASAT, ALAT, GGT or albumin) to decipher a possible connection between HERV-K(HML-2) expression and diminished liver function. Interestingly, increased HERV-K(HML-2) transcript levels were clearly associated with the increase in non-invasive blood makers, as for example ASAT, ALAT, or albumin levels, which are all indicators of impaired liver synthesis function as well as worse disease prognosis. Moreover, the three patients who failed to achieve DAA treatment success were associated with higher HERV-K(HML-2) transcript levels compared to the rest of the cohort. Together these findings provide evidence that HERV-K(HML-2) transcript levels might serve as a marker for the severity of liver damage in hepatitis C patients.

It is well known that HCV-infected patients often develop liver cirrhosis, which can ultimately lead to liver cancer as well as other cancers or autoimmune diseases, which stay even after viral clearance using DAA treatment [40,41,42]. This raises the question whether reverse transcriptase inhibitors should be included in HCV antiviral therapy to improve treatment success. Reverse transcriptase inhibitors such as Raltegravir are well known and in clinical use to treat retroviral diseases such as human immunodeficiency virus (HIV) [43]. There is also evidence that reverse transcriptase as well as integrase inhibitors used in HIV treatment can inhibit human endogenous retroviruses [44]. Importantly, clinical trials are already ongoing to test if suppression of the HERV-K(HML-2) *env* protein using antiretroviral drugs [45] influences the neurophysiological outcomes of amyotrophic lateral sclerosis (ALS) symptoms, as the overexpression of the HERV-K(HML-2) *env* protein has been proposed as a possible causative factor in patients with ALS [19].

Overall, our data indicate a connection between HERV-K(HML-2) expression and HCV-associated liver cirrhosis. However, it is unclear if increased HERV-K(HML-2) transcript levels contribute to the development of HCV pathologies or if their elevated expression results from a secondary side effect. Nevertheless, our data clearly highlight the need to understand the functional role of HERVs in the development of HCV pathogenesis to improve antiviral treatment.

## Figures and Tables

**Table 1:** Drug combinations among the cohort of hepatitis C patients.

Drug Combinations	Number of Patients
Simeprevir/Sofosbuvir/Ribavirin	5
Simeprevir/Sofosbuvir	6
Daclatasvir/Sofosbuvir	16
Daclatasvir/Sofosbuvir/Ribavirin	3
Ledipasvir/Sofosbuvir	5
Ledipasvir/Sofosbuvir/Ribavirin	2
Viekirax/Exviera	4
Viekirax/Ribavirin	1

**Table 2:** Correlation of HERV-K(HML-2) transcript levels with mean albumin levels over the course of DAA treatment.

Mean Albumin Levels			
Time Point	HERV-K(HML-2) < 1	HERV-K(HML-2) > 1	*p*-Value
baseline	38.04 g/L	32.10 g/L	*p* = 0.005
week 2	37.52 g/L	31.89 g/L	*p* = 0.003
week 4	38.45 g/L	32.56 g/L	*p* = 0.001
end of treatment (EOT)	38.35 g/L	34.10 g/L	*p* = 0.004
SVR 12	38.68 g/L	35.30 g/L	*p* = 0.048

## Data Availability

Data generated in this study are available from the corresponding author Michelle Vincendeau.
